# BRD9 Inhibition, Alone or in Combination with Cytostatic Compounds as a Therapeutic Approach in Rhabdoid Tumors

**DOI:** 10.3390/ijms18071537

**Published:** 2017-07-16

**Authors:** Katja F. Krämer, Natalia Moreno, Michael C. Frühwald, Kornelius Kerl

**Affiliations:** 1 University Children’s Hospital Muenster, Department of Pediatric Hematology and Oncology, 48149 Münster, Germany; k_krae03@uni-muenster.de (K.F.K.); Natalia.MorenoGalarza@ukmuenster.de (N.M.); 2 Children’s Hospital and Swabian Children’s Cancer Center, 86156 Augsburg, Germany; michael.fruehwald@klinikum-augsburg.de

**Keywords:** rhabdoid tumor, BRD9, SWI/SNF complex, targeted therapy

## Abstract

Rhabdoid tumors (RT) are malignant neoplasms of early childhood. Despite intensive therapy, survival is poor and new treatment approaches are required. The only recurrent mutations in these tumors affect *SMARCB1* and less commonly *SMARCA4*, both subunits of the chromatin remodeling complex SWItch/Sucrose Non-Fermentable (SWI/SNF). Loss of these two core subunits alters the function of the SWI/SNF complex, resulting in tumor development. We hypothesized that inhibition of aberrant SWI/SNF function by selective blockade of the BRD9 subunit of the SWI/SNF complex would reduce tumor cell proliferation. The cytotoxic and anti-proliferative effects of two specific chemical probes (I-BRD9 and BI-9564) which target the bromodomain of SWI/SNF protein BRD9 were evaluated in 5 RT cell lines. Combinatorial effects of I-BRD9 and cytotoxic drugs on cell proliferation were evaluated by cytotoxicity assays. Single compound treatment of RT cells with I-BRD9 and BI-9564 resulted in decreased cell proliferation, G1-arrest and apoptosis. Combined treatment of doxorubicin or carboplatin with I-BRD9 resulted in additive to synergistic inhibitory effects on cell proliferation. In contrast, the combination of I-BRD9 with vincristine demonstrated the antagonistic effects of these two compounds. We conclude that the BRD9 bromodomain is an attractive target for novel therapies in this cancer.

## 1. Introduction

Rhabdoid tumors (RT) are highly aggressive malignancies with an age peak in children younger than three years [1]. They are commonly localized in the brain (atypical teratoid/rhabdoid tumor, AT/RT), the kidney (rhabdoid tumor of the kidney, RTK) and in soft tissues (malignant rhabdoid tumor, MRT). Intensive, multimodal treatment approaches have improved the clinical outcome of these young patients in a stepwise manner. However, their prognosis remains dismal and the median duration of survival in clinical studies still does not exceed 9 to 17 months (AT/RT) [2]. Unfortunately, intensification of treatment has resulted in increased therapy-associated mortality but not further improved prognosis [3]. Thus, new therapeutic strategies are urgently needed. 

RT are genetically characterized by bi-allelic loss of *SMARCB1* (SWI/SNF Related, Matrix Associated, Actin Dependent Regulator Of Chromatin, Subfamily B, Member 1) (98% of RT) or *SMARCA4* (2% of RT), which are subunits of the chromatin remodeling complex SWI/SNF (SWItch/Sucrose Non-Fermentable) [4,5,6,7]. The SWI/SNF complex consists of one ATPase subunit, which is either BRM or BRG1, and different variable subunits. Three essential core subunits have been identified: BAF155, BAF170 and BAF47 (SMARCB1) [8]. Depending on cell differentiation and environmental factors, the SWI/SNF complex can include 7 to 15 additional subunits [9]. This complex uses energy from ATP hydrolysis to interact with chromatin and influences the bond of DNA to histones [10]. By its chromatin remodeling function the SWI/SNF complex controls the expression of multiple genes and is involved in cellular processes such as stem cell differentiation and development [11]. Loss of SWI/SNF subunits leads to deregulation of tumor-associated pathways such as Wnt/β-catenin or Hedgehog/GLI [12,13] and deregulation of epigenetic modulators such as histone deacetylases (HDAC) and Enhancer of Zeste Homolog 2 (EZH2) [7,14].

A largely unstudied subunit of the SWI/SNF complex is the bromodomain containing protein 9 (BRD9) [15]. Bromodomain containing proteins recognize acetylated lysine residues on histones and are involved in epigenetic mechanisms such as regulation of transcription, chromatin remodeling and histone modification [16,17]. BRD9 is also discussed as a reader of butyryl lysines, but its specific function remains unknown [18]. Histone acetylases (HAC) and HDACs catalyze acetylation and deacetylation of lysine residues of histones and other proteins [19]. Acetylation mainly results in a loose chromatin structure and enhanced accessibility of the DNA facilitating transcription [20]. By reading these epigenetic codes, BRD9 might be involved in SWI/SNF-associated gene transcription, DNA repair and cell differentiation. In acute myeloid leukemia (AML) cells, BRD9 depletion resulted in G1 arrest [21]. Mutations of BRD9 and five other SWI/SNF complex related proteins are associated with a higher number of overall genetic alterations and genomic instability in lung cancer [22]. 

Based on the hypothesis that tumorigenesis by RTs may not be driven by complete loss of SWI/SNF function, but rather an aberrant activity of the remaining complex [23], we investigated the effects of selective inhibition of BRD9 on RT growth in vitro. Here we demonstrate for the first time that inhibition of BRD9 by small chemical compounds, alone or in combination with cytotoxic compounds, affects cell proliferation, cell viability and cell cycle progression of RT cells. As subunits of the SWI/SNF complex are altered in approximately 20% of all neoplasms, this data might be the basis for targeted approaches not only in RT but also in other tumor entities [15,24].

## 2. Results

### 2.1. Small-Molecule BRD9 Inhibitors Decrease Rhabdoid Tumor Cell Proliferation In Vitro

To evaluate whether inhibition of the SWI/SNF subunit BRD9 blocks proliferation of RT cells, five RT cell lines derived from tumors of different anatomic localization (BT12, BT16, Chla266 are of intracranial and G401, KD are of extracranial RT origin) were incubated in the presence of two available small-molecule BRD9 inhibitors (BRD9i), BI-9564 and I-BRD9. These molecules were originally developed to target the acetyl-lysine binding domain (bromodomain) of BRD9. Both inhibitors have a high potency and selectivity against BRD9 [25,26]. They target BRD7 to a lesser extent, but present a very low or no affinity for Bromodomain and extra-terminal (BET) family members.

**Figure 1:**
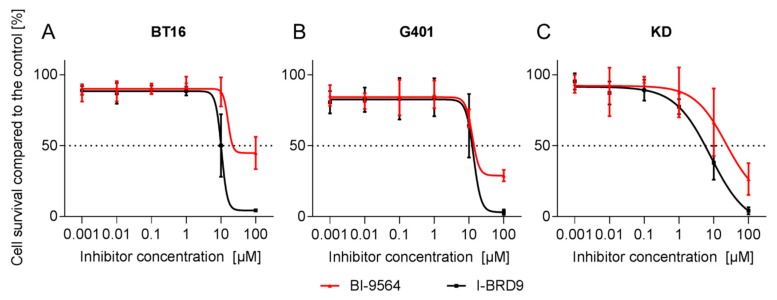
Proliferation curves of rhabdoid tumor (RT) cell lines BT16 (**A**), G401 (**B**) and KD (**C**) incubated for 72 h at different BRD9 inhibitors tested, BI-9564 (red) or I-BRD9 (black) concentrations based on MTT proliferation assays. Dotted line indicates 50% cell survival compared to the control. (*n* ≥ 3; data are presented in means ± SD).

Effects of the two inhibitors on cell proliferation were evaluated after 72 or 144 h of incubation at increasing concentrations in MTT (3-(4,5-dimethylthiazol-2-yl)-2,5-diphenyl-2*H*-tetrazolium bromide) proliferation assays. Both BRD9i had negative effects on the proliferation of all RT cell lines (Figure 1). Half-maximal inhibitory concentrations (IC_50_) at 72 h ranged from 8.1 µM to 22.3 µM for both BRD9i. In general, IC_50_ values were lower for both inhibitors after six days (144 h) than after three days (72 h) (Table 1). In BT12 and Chla266 cell lines, an anti-proliferative effect could be observed only after longer incubation time (144 h).

### 2.2. BRD9 Inhibitors Induce G1 Cell Cycle Arrest in RT Cell Lines

**Figure 2:**
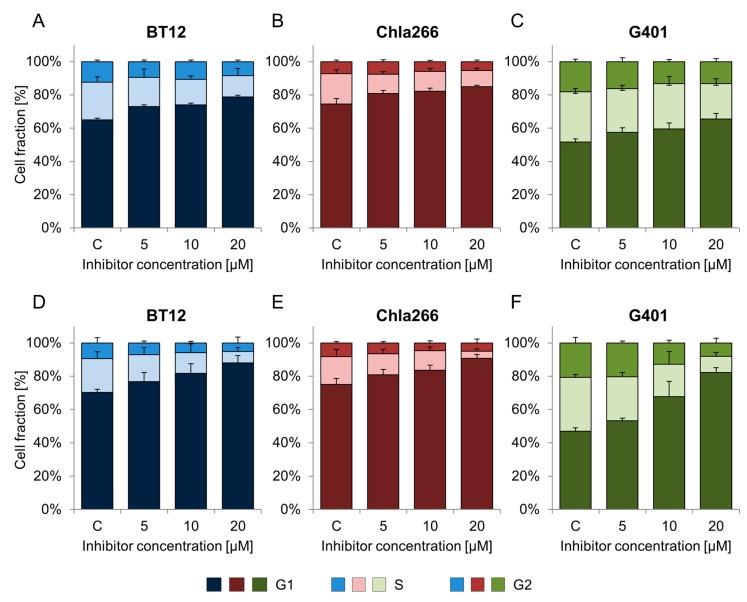
Impact of BRD9 inhibitors on cell cycle in RT. Different RT cell lines were treated with BI-9564 (**A**–**C**) or I-BRD9 (**D**–**F**) inhibitors in a range of 5 to 20 µM and incubated for 72 h. Cell cycle profiles, defined by G1, S and G2 phases, of BT12 (**A**,**D**), Chla266 (**B**,**E**) and G401 (**C**,**F**) cell lines are shown. (c = control; *n* ≥ 3; error bars indicate SD).

To determine the effects of BI-9564 and I-BRD9 on cell cycle progression, cells were treated with increasing concentrations of the inhibitors and then analyzed by flow cytometry. The G1 phase population significantly increased in a dose-dependent manner in RT cell lines treated with the substances (*p* < 0.05, one-way ANOVA (analysis of variance)). The strongest impact was observed for I-BRD9 (20 µM) on G401, but also the AT/RT cell lines BT12 and Chla266 were arrested in cell cycle phase G1 (Figure 2). These data were confirmed by the second BRD9i: G401, BT12 and Chla266 cells lines showed G1 arrest following BI-9564 treatment (Figure 2 and Table 2).

### 2.3. Treatment with BRD9 Inhibitors Reduces the Viability of RT Cells In Vitro

**Figure 3:**
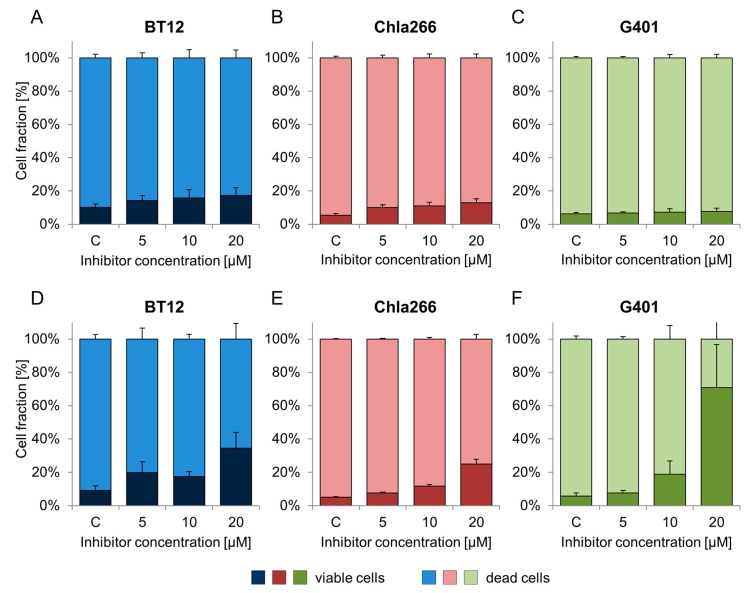
Dose-dependent effects of BI-9564 (**A**–**C**) and I-BRD9 (**D**–**F**) on cell viability of BT12 (**A**,**D**), Chla266 (**B**,**E**) and G401 (**D**,**F**) after 72 h. (c = control; *n* ≥ 3; error bars indicate SD).

In order to examine whether the observed anti-proliferative effect was accompanied by cell death, apoptosis assay was performed. BRD9i decreased the viability of the cells in a dose-dependent manner (Figure 3). I-BRD9 treatment showed a significant impact on cell viability of BT12, Chla266, and G401 cells at 20 µM (Table 3). In G401, the percentage of dead cells increased by 65.1 ± 24.1% compared to the control (20 µM) (Table 3). The fraction of dead AT/RT cells grew in a dose-dependent way from 9.1% (control) to 35% (20 µM) in BT12 cells and from 5% (control) to 12% (10 µM) to 25% (20 µM) in Chla266 cells (Figure 3D–F; Table 3).

BI-9564 significantly changed cell viability in Chla266 treated cells with 5, 10, or 20 µM. In BT12 and G401 cell lines minor changes were observed (Figure 3A–C; Table 3).

### 2.4. I-BRD9 Synergistically Inhibits RT Cell Growth in Combination with Carboplatin and Doxorubicin

**Figure 4:**
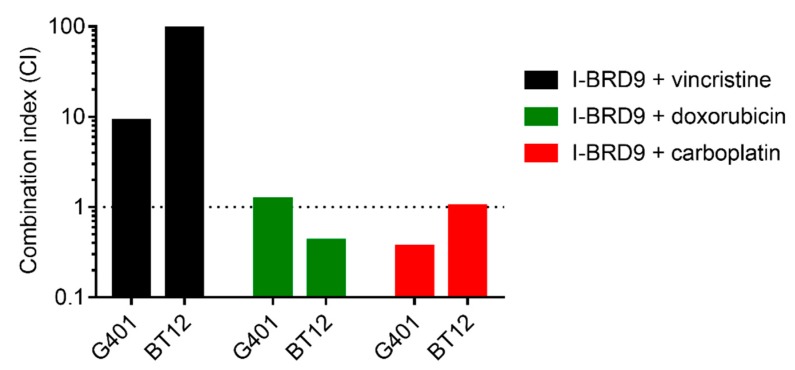
Combination indices (CI) of I-BRD9 combined with carboplatin, doxorubicin or vincristine. Data from cytotoxicity assays were analyzed by median effect method of Chou and Talalay. Dotted line marks CI = 1. CI < 1 indicates synergism, CI > 1 antagonism of combined drugs. (*n* ≥ 3).

Chemotherapy is an important element in the therapy of RT patients. Vincristine, doxorubicin and carboplatin are commonly used cytotoxic drugs in these kind of neoplasms [27]. To quantify the effects of BRD9i treatment in combination with chemotherapy, viability assays were performed and data were analyzed by the median effect method by Chou and Talalay [28]. Our results show that cells simultaneously treated with I-BRD9 and with carboplatin or doxorubicin proliferated less than cells receiving monotherapy at equivalent doses. The combination indices calculated after analysis of the obtained data for I-BRD9 and carboplatin showed synergistic or additive effects on cell growth inhibition in BT12 and in G401 cell lines, respectively. I-BRD9 combined with doxorubicin decreased BT12 proliferation in a synergistic way, but did not show a cooperative effect on G401 growth inhibition. Similarly, contemporaneous treatment with vincristine and I-BRD9 exerted antagonistic effects on tumor cell proliferation (CI > 1) (Figure 4 and Table 4). 

## 3. Discussion

Research approaches over the past years have focused on targeting deregulated pathways in RT [29]. Different signaling pathways (WNT, SHH, FGFR and many more) as well as epigenetic modifiers (HDAC, EZH2) have been described as being deregulated in these kind of tumors [12,13,14,30,31]. Several targeted compounds show promising preclinical results and some of them are tested in early clinical studies such as CDK4/6 inhibitors, EZH2 inhibitors and Aurora kinase inhibitors, with disappointing to discordant responses [29,32,33,34]. In this project, we hypothesized a hierarchical model of events resulting in RT-genesis: One genetic alteration (*SMARCB1/SMARCA4* mutation) leads to the deregulation of diverse epigenetic modulators (HDACs, EZH2 or DNA-methyltransferases) which in turn deregulate distinct signaling pathways. Following this model and also being aware that RT are divided into several subgroups by the deregulation of distinct signaling pathways [6], we hypothesized that inhibition of BRD9 might be a promising targeted approach for all RT, independent of their molecular subgroups. 

Two BRD9 inhibitors were used to provide first evidence for this hypothesis: BI-9564 and I-BRD9, which were developed independently following a structure based design [25,26]. Both compounds are highly selective for BRD9. For instance, BI-9564 shows no relevant activity against BET members below 100 µM (α-assay) [25] and I-BRD9 has 700 fold higher selectivity for BRD9 than for other BET family members [26]. 

Bromodomain containing proteins are divided into two subfamilies by their structural domains: BET and non-BET [16]. BET proteins directly influence transcription and cell cycle progression [16,35]. In the last years, several inhibitors, mainly against BET proteins, were developed and showed anticancer effects in vitro and in vivo [36]. Currently, some of them are being tested for solid and hematopoietic malignancies in clinical trials [37].

BRD9 inhibition is not yet well described in the literature. Recently, it was shown that the *knockout* of BRD9 as well as inhibition by BRD9 inhibitors results in G1 arrest in AML cell lines, but does not induce apoptosis [21]. Inhibition by BI-9564 blocked proliferation of AML cells [25]. In a mouse model of AML, BI-9564 significantly reduced tumor growth and improved survival in treated mice [25]. BRD9 inhibition by I-BRD9 in Kasumi-1 cells resulted in lower expression of various cancer- associated genes (*CLEC1*, *DUSP6*, *FES* and *SAMSN1*). DUSP6 deletion sensitizes to various cytotoxic agents and is involved in the DNA damage repair [38]. It influences MAP kinases by dephosphorylation [39]. In breast cancer the suppression of DUSP6 leads to reduced cell proliferation and results in G0/G1 arrest [40]. *FES* is discussed as a proto-oncogene as well as a tumor suppressor [41]. This tyrosine kinase plays a crucial role in tumor microenvironment, cell-cell interaction, organization of the cytoskeleton and signal transduction [42,43]. SAMSN1 regulates HDAC1 activity and is known as a tumor suppressor in multiple myeloma [44,45]. Its downregulation is related to a poor prognosis in gastric cancer and hepatocellular carcinoma patients [46,47].

Treatment of RT cell lines with BI-9564 and I-BRD9 results in G1-arrest and apoptosis. In line with these results, recent publications support these data by showing that the tumor proliferation of SMARCB1 negative tumors is dependent on the residual activity of the SWI/SNF complex [23].

The mechanism how BRD9i might induce G1 arrest in RT is not clear. In AML cells knockout of BRD9 as well as inhibition by I-BRD9 leads to downregulation of Myc and downstream elements [21]. SMARCB1 negatively regulates Myc resulting in an overexpression of Myc in SMARCB1 deficient cells [48]. Further investigation is required to prove whether Myc expression is the link between BRD9 inhibition and G1 arrest in RT.

As single targeted treatment approaches often cause secondary resistances, we investigated combinatorial effects of chemotherapy plus I-BRD9. For these approaches, chemotherapeutic compounds commonly applied to children with RT, were used: vincristine, doxorubicin and carboplatin.

Vincristine is a highly cytotoxic vinca alkaloid. Aside from other mechanisms leading to apoptosis, vincristine binds to tubulin and inhibits mitosis [49] leading to G2/M cell cycle arrest. In our experiments, vincristine and BRD9i showed antagonistic effects on RT proliferation. This antagonism might be explained by the arrest of RT cells treated with BRD9i in G1 phase, preventing them from entering G2/M phase, in which vincristine exerts its function.

Doxorubicin is a potent anthracycline compound and an important part of RT treatment [27]. It intercalates with the DNA and inhibits topoisomerase II. The use of this highly effective drug is mainly limited by cardiotoxicity resulting in dilative cardiomyopathy [50]. Mutations in various subunits of the SWI/SNF complex sensitize for chemotherapy with doxorubicin and cisplatin in yeast [51]. In contrast, mutations in SNF5, the yeast homolog of SMARCB1, resulted in reduced sensitivity to doxorubicin [52]. According to our results, BRD9i treatment sensitizes RT cell lines to doxorubicin.

Carboplatin is a platinum based drug which crosslinks the DNA double strands [53]. It is widely used in high dose chemotherapy protocols but the applicable dose is limited by severe myelosuppression [53]. In our combined treatment approach, I-BRD9 and carboplatin cooperatively inhibited cell proliferation.

This would be in accordance to published data where downregulation of BRG1 in lung cancer leads to higher sensitivity to cisplatin by disturbing the repair of induced DNA lesions [54].

In summary, we provide preclinical data which show a promising targeted directed approach of BRD9i in combination with chemotherapy for RT treatment. Furthermore, the effects of each chemotherapeutic compound in combination with BRD9i should urgently be re-evaluated, since drugs like vincristine, have antagonistic effects. BRD9 inhibitors could be utilized in specific therapy cycles in addition to the established drugs.

In the future, preclinical experiments have to clarify the mechanism of action of BRD9-inhibitors and will extend these observations to in vivo testing. Inhibiting further subunits of the SWI/SNF complex might additionally enhance the vulnerability of RT cells to chemotherapy. Combined approaches including BRD9i might allow reducing severe adverse effect by decreasing chemotherapy dosage in RT patients. And as the SWI/SNF complex is mutated in 20% of all cancers, our results might be extended to other SWI/SNF related tumor entities.

## 4. Materials and Methods

### 4.1. Cell Culture

G401 (RT of the kidney) and KD (RT of the soft tissue) were maintained in Dulbeccos Modified Eagles Medium-high glucose (DMEM) (Sigma Aldrich, St. Louis, MI, USA) supplemented with 10% fetal bovine serum (FBS) (Biochrom, Merck, Darmstadt, Germany), BT16 (AT/RT) in DMEM with 17% FBS. For BT12 (AT/RT) and Chla266 (AT/RT) we used Iscove’s Modified Dulbecco’s Medium (IMDM, Gibco, Gaithersburg, MD, USA) supplemented with 20% FBS (South America origin, Gibco, Gaithersburg, MD, USA). All media contained 1% Penicillin/Streptomycin (Gibco, Gaithersburg, MD, USA). Cells were incubated at 37 °C at 5% CO_2_.

### 4.2. BRD9 Inhibitors and Cytostatics

I-BRD9 (Tocris Bioscience, Bristol, UK) and BI-9564 (Tocris Bioscience, Bristol, UK) were dissolved in Dimethyl Sulfoxide (DMSO) (AppliChem, Hannover, Germany) as stock solutions of 10 mM and aliquoted to only freeze thaw once.

The cytostatic compounds carboplatin, doxorubicin and vincristine were provided by the pharmacy of the Department of pediatric hematology and oncology, University Children’s Hospital Muenster.

### 4.3. Cytotoxicity Assay

Cells were seeded as suspensions as follows: BT16, G402, KD: 3000 cells/50 µL, BT12, Chla266: 5000 cells/50 µL, into 96-well-plates. 50 µL of medium containing the drugs at different concentrations were added on top after 24 h. I-BRD9 and BI-9564 were tested in final concentrations ranging from 0.001 to 100 µM and cytostatic drugs from 0.0001 µM to 10 µM. For the combined therapy approaches, we used I-BRD9 and cytostatic substances in a ratio of 10:1 respectively. After 72 h or 144 h, 10 µL of MTT reagent (Merck, Darmstadt, Germany) were added per well. Metabolic active cells reduce yellow tetrazolium salt to purple formazan crystals. Those were dissolved with isopropanol- /HCl 0.04 N after 3 h and a color change from yellow to purple was observed. Samples were spectrophotometrically evaluated on a Multiskan Ascent multiplate reader (Labsystems, Helsinki, Finland) at 570 nm and 630 nm and a baseline was calculated. Assays running for six days were provided with fresh medium and drugs on day three. All assays were performed in technical triplicates and at least three independent times. Data was analyzed using GraphPad Prism software version 6.0.

Combined drug effects on cell proliferation were evaluated by the median effect method by Chou and Talalay [28] adapted to the MTT assay data (Microsoft Excel 2010). CI values > 1 indicate antagonism, CI values < 1 synergism of the combined drugs.

### 4.4. Apoptosis Assay and Cell Cycle Analysis

One milliliter cell suspension of G401 (10,000 cell/mL), BT12 (30,000 cell/mL) and Chla266 (30,000 cell/mL) was seeded per well into 12-well plates. On the next day, medium was replaced by medium containing I-BRD9 or BI-9564 at a final concentration of 0 µM, 5 µM, 10µM or 20 µM and cells were incubated for 72 h. After this time, cells were collected and washed with PBS. Induction of apoptosis following the treatments was detected by staining the cells with FITC-Annexin V Apoptosis Detection Kit (BD Bioscience, San Jose, CA, USA) as follows: cells were stained with FITC Annexin V and propidium iodide. After incubating in the dark for 15 min at room temperature, cells were analyzed by Flow Cytometry (FACS CantoII). Data were analyzed using FlowJo (Tree Star Inc., Ashland, OR, USA).

For cell cycle, 100 μL of cell suspension were incubated with 4′,6-diamidino-2-phenylindole (DAPI, DAPI powder, Applichem, Hannover, Germany) and measured using FACS CantoII flow cytometry system. Data from cell cycle analysis were analyzed using FlowJo (Tree Star Inc., Ashland, OR, USA). The calculation of the area under the curve during cell cycle analysis was achieved using the Watson- or Dean-Jett-Fox-models on all samples of a particular cell line.

### 4.5. Statistical Analyses

All Data are represented as mean values ± SD. For comparison of more than 2 values ANOVA One-way test was used. All statistical analyses were performed using Graph Pad Prism 6.0 software. Significance was assumed when *p* < 0.05.

## Figures and Tables

**Table 1:** Half maximal inhibitory concentrations (IC_50_) of BI-9564 and I-BRD9 in RT cell lines incubated for 72 or 144 h. Cell proliferation was evaluated by MTT cytotoxicity assays. (*n* ≥ 3).

IC_50_ (µM)	BI-9564		I-BRD9	
Cell Line	72 h	144 h	72 h	144 h
BT12	>100	17.2	21.2	8.2
BT16	15.9	-	11.2	-
Chla266	>100	33.7	13.3	24.7
G401	12.5	10.8	13.4	6.1
KD	22.3	10.8	8.1	7.1

**Table 2:** Percentage of cells in G1 cell cycle phase after incubating with BI-9564 and I-BRD9 at the indicated concentrations for 72 h. (*n* ≥ 3; mean ± SD; * treatment vs. control with *p* < 0.05, one-way ANOVA).

Treatment		Cell Line		
		BT12	Chla266	G401
BI-9564	C	65.0 ± 3.9	74.1 ± 3.2	51.7 ± 2.0
	5 µM	73.1 ± 4.6	80.0 ± 1.8	57.6 ± 2.6
	10 µM	74.0 ± 3.4	81.2 ± 1.8 *	59.5 ± 3.6 *
	20 µM	78.9 ± 6.3 *	83.6 ± 0.9 *	65.5 ± 3.3 *
I-BRD9	C	69.0 ± 1.9	74.2 ± 3.6	46.7 ± 1.9
	5 µM	75.0 ± 5.3	79.5 ± 3.2	53.0 ± 1.6
	10 µM	80.1 ± 5.8	82.7 ± 2.9 *	67.1 ± 9.1 *
	20 µM	85.6 ± 4.3 *	89.3 ± 2.5 *	78.1 ± 2.7 *

**Table 3:** Percentage of dead RT cells after incubation with different BI-9564 and I-BRD9 concentrations for 72 h. (*n* ≥ 3; mean ± SD, * treatment vs. control with *p* < 0.05, one-way ANOVA).

Treatment		Cell Line		
		BT12	Chla266	G401
BI-9564	C	10.1 ± 2.2	5.4 ± 1.0	6.2 ± 0.8
	5 µM	14.2 ±3.0	10.0 ± 1.7 *	6.8 ± 0.8
	10 µM	15.9 ± 4.9	11.0 ± 2.3 *	7.3 ± 2.0
	20 µM	17.3 ± 4.7	12.9 ± 2.4 *	7.6 ± 2.1
I-BRD9	C	9.1 ± 2.8	5.0 ± 0.3	5.7 ± 1.9
	5 µM	19.9 ± 6.6	7.7 ± 0.5	7.7 ± 1.5
	10 µM	17.6 ± 2.8	11.7 ± 1.0 *	18.8 ± 8.1
	20 µM	34.6 ± 9.4 *	25.0 ± 2.9 *	70.8 ± 26.0 *

**Table 4:** IC_50_ and combination indices (CI) of combined treatments. Treatment of BT12 and G401 with combinations of I-BRD9 and three cytotoxic drugs for 72 h. CI < 1 indicates synergistic effects and CI > 1 antagonistic effects of combined drugs. *R*^2^ represents the determination coefficient of linear regression in median effect plot. (*n* ≥ 3).

Treatment	BT12			G401		
	IC_50_ (µM)	CI	*R*^2^	IC_50_ (µM)	CI	*R*^2^
Carboplatin + I-BRD9	40.9	1.01	0.83	9.1	0.37	0.94
Doxorubicin + I-BRD9	0.94	0.42	0.94	0.096	1.2	0.84
Vincristine + I-BRD9	16.7	96.1	0.59	0.008	9.0	0.88
